# "We're not short of people telling us what the problems are. We're short of people telling us what to do": An appraisal of public policy and mental health

**DOI:** 10.1186/1471-2458-8-314

**Published:** 2008-09-15

**Authors:** Mark Petticrew, Stephen Platt, Allyson McCollam, Sarah Wilson, Sian Thomas

**Affiliations:** 1 Public and Environmental Health Research Unit, London School of Hygiene and Tropical Medicine, London, WC1E 7HT, UK; 2 Research Unit in Health, Behaviour and Change (RUHBC), University of Edinburgh, EH8 9AG, UK; 3 Scottish Development Centre for Mental Health, Edinburgh, EH6 5QN, UK; 4 Sociology, School of Social and Political Studies, University of Edinburgh, Edinburgh, EH8 9LL, UK; 5 Medical Research Council Social and Public Health Sciences Unit, University of Glasgow, Glasgow, G12 8RZ, UK

## Abstract

**Background:**

There is sustained interest in public health circles in assessing the effects of policies on health and health inequalities. We report on the theory, methods and findings of a project which involved an appraisal of current Scottish policy with respect to its potential impacts on mental health and wellbeing.

**Methods:**

We developed a method of assessing the degree of alignment between Government policies and the 'evidence base', involving: reviewing theoretical frameworks; analysis of policy documents, and nineteen in-depth interviews with policymakers which explored influences on, and barriers to cross-cutting policymaking and the use of research evidence in decisionmaking.

**Results:**

Most policy documents did not refer to mental health; however most referred indirectly to the determinants of mental health and well-being. Unsurprisingly research evidence was rarely cited; this was more common in health policy documents. The interviews highlighted the barriers to intersectoral policy making, and pointed to the relative value of qualitative and quantitative research, as well as to the imbalance of evidence between "what is known" and "what is to be done".

**Conclusion:**

Healthy public policy depends on effective intersectoral working between government departments, along with better use of research evidence to identify policy impacts. This study identified barriers to both these. We also demonstrated an approach to rapidly appraising the mental health effects of mainly non-health sector policies, drawing on theoretical understandings of mental health and its determinants, research evidence and policy documents. In the case of the social determinants of health, we conclude that an evidence-based approach to policymaking and to policy appraisal requires drawing strongly upon existing theoretical frameworks, as well as upon research evidence, but that there are significant practical barriers and disincentives.

## Background

There is sustained interest in assessing the impacts of policies outside the health sector on health and health inequalities. Current approaches include collecting new outcome data as part of new evaluation studies, and health impact assessment, which emphasises the prospective estimation of positive and negative impacts of new policies, and the importance of which has been emphasised in documents published by national government and international organisations such as EU and WHO [1-4]. Similar points have been made many times subsequently [5-7]. Policy impact assessment itself is not new, of course [8-10]. However interest in the need to assess the effects of policies has been stimulated further in the past ten years by debates about the need for 'evidence-based' health policy. This is often taken to mean the informed, explicit and transparent use of robust scientific evidence in informing policy decisions, with reference to an existing evidence base [11,12].

The conceptual and methodological roots of such approaches are widespread, drawing on economics, epidemiology, risk assessment, environmental and other forms of risk assessment, sociology, politics and other disciplines [2]. Policy appraisal and health impact assessment, methods, for example, similarly draw on all these disciplines, including in-depth quantitative and qualitative research, and 'tool-kit' – based approaches, though these have come in for criticism as they place too little emphasis on the soundness of the evidence upon which they draw [13].

We were able to consider some of these issues in a project commissioned by NHS Health Scotland, which involved carrying out an appraisal of current Scottish policy with respect to its potential (direct, or indirect) effects on mental health. It required devising a method of assessing the degree of alignment between policies and the 'evidence base'. We developed an approach which involved documentary analysis (using Scottish Government policy documents as data) and in-depth interviews with policymakers to explore the role of research evidence in the Scottish Government policymaking process. This paper describes the theory and methods of this project, and presents findings from both the documentary analysis and the qualitative interviews. First, however, a brief description of the context of the project is warranted.

### The Scottish policy context

Mental health improvement is one of three integral components of the Scottish Government's agenda for health improvement. In October 2001 the National Programme for Improving Mental Health and Well-being was launched to raise the profile of, and to support further action in, mental heath improvement. Its aims were to raise awareness and promote mental health and well-being, to eliminate stigma and discrimination, to prevent suicide, and to promote and support recovery. It also acknowledged that the solutions lay not just within the health sector but required a response from other Government departments.

In line with this, the National Programme aimed to contribute to the development of "mentally healthy public policy... creating supportive environments, improving access to services and supports (particularly for marginalised and disadvantaged groups), strengthening community action, supporting community-led mental health initiatives, developing and consolidating local community partnerships and enhancing the role and contribution of community development and community education and learning". It also spoke of the need to improve mental health and wellbeing through good quality housing, quality built environments, environmental policies, transport infrastructure, policing, and health and social care services, and other public services. These ambitious goals reflected a wider recognition within the Scottish Government that many of its key objectives involved tackling complex social and economic issues which require a multi-agency cross-cutting response, with an increase since 1997 in the number of cross-cutting initiatives [14].

To this end the research reported in this paper was commissioned to appraise Scottish Government policy with regards to the links between the evidence base on the (likely) impact of mental health improvement policies, on the one hand, and the content of 'active' Scottish Government policy statements/documents, on the other.

Among the objectives of the project were:

1. To map out the key policy areas in Scotland;

2. To gather key policy documents and identify references to mental health improvement;

3. In each policy area to identify what existing research evidence tells us about the (potential) mental health impacts (direct, or indirect), and

4. To assess the degree to which current policy and the evidence are aligned.

'Improving mental health' was defined as promoting mental health in the whole population (for example, self esteem and confidence, feelings of belonging, coping skills, resilience, among others), preventing mental health problems, and improving the quality of life of people experiencing mental health problems.

## Methods

We developed an approach (described below) that was rooted in a specific consideration of the determinants of mental health inequalities. In addition, investigating some of the issues around using public policy to improve mental health and wellbeing, and the barriers to this, required an in-depth approach and thus qualitative interviews were also felt to be necessary.

There were five main stages to the project: first, selection of policies for appraisal; second, developing a theoretical model; third, mapping the evidence base; fourth, policy document analysis; and, finally, in-depth interviews with policymakers. The methods adopted at each stage are described below.

### 1. Selection of policies

First, the list of policy documents for analysis was agreed with commissioners (See list in Table 2).

**Table 2:** Scottish Government policy documents: the sample (government department/area in brackets)

*A Scotland where everyone matters *(Social Inclusion)
*Being well – doing well *(Education)
*It's everyone's job to make sure I'm alright *(Education)
*For Scotland's children *(Education)
*A smart, successful Scotland *(Enterprise, Transport, Lifelong Learning)
*The way forward: framework for economic development in Scotland *(Enterprise, Transport, Lifelong Learning)
*Life through learning through life *(Enterprise, Transport, Lifelong Learning)
*Scotland's transport *(Enterprise, Transport, Lifelong Learning)
*National plan on alcohol problems *(Justice)
*Towards a healthier Scotland *(Health)
*Improving health in Scotland – the challenge *(Health)
*The equality strategy *(Equalities)
*Race equality scheme *(Equalities)
*Community regeneration statement *(Community regeneration)
*Homelessness task force final report *(Homelessness)
*Housing improvement task force *(Housing)
*Scottish compact *(Voluntary issues)
*Cultural policy statement *(Culture, Arts and Sport)
*Let's make Scotland more active *(Culture, Arts and Sport)
*Rural Scotland *(Rural Development)

### 2. Developing a theoretical model

Next, it was essential to review the causal pathways by which mental health outcomes might be expected to be generated. This was intended to provide a guide to the identification of those effects when examining the policy documents and conducting the interviews. From our own knowledge and contacts we therefore identified several relevant frameworks and sources [15-19]. From these we produced a list of key policy-relevant social determinants (Table 1). We used this list (with overlaps removed) to produce, first, a list of policy-relevant influences which appeared in one or more existing models of mental health determinants, and, second, a preliminary list of some of the main sources of evidence. Guided by this new framework we then carried out a rapid review to map the evidence base on the social determinants of population mental health and well-being.

**Table 1:** Potential determinants of mental health identified from previous frameworks

**STAKES **[16]
Family sphere
School
Work
Community and environment
Administration and services

*Societal structures and resources*
Societal policies
Organisational policies
Educational resources
Housing resources
Economic resources
Availability and quality of services

*Cultural values*
Prevailing societal values (equity, human rights)
Societal value given to mental health
Rules regulating social interactions
Social criteria of mental health and ill-health

**Friedli (2003) **[17]
Health, and Mental health and social services
Neighbourhood (as opposed to housing)
Quality of the natural environment
Environment (including noise, pollution)
Cultural and leisure facilities (including sport)
Community safety
Childcare and self-help networks

***The Solid Facts ****(Wilkinson & Marmot, 2003) *[18]
'Stress' generated by institutions such as schools and workplaces
Social isolation
Material and financial insecurity
Poor circumstances during pregnancy
Discrimination
Racism
Prisons
Addiction
Transport (no explicit mental health links)

***OTHER MODELS ****(e.g., Dahlgren and Whitehead) *[19]
Biology and genetic endowment (not directly susceptible to policy intervention)
Gender
Water and sanitation

### 3. Mapping the evidence base

Within the limited available time and resources we did not aim to review all existing literature on the determinants of mental health and mental health inequalities, or on the effectiveness of interventions to address these. Instead, we relied mainly on secondary sources [20-22]. Within the available time and resources we did not aim to review all existing literature nor was this the purpose of the project. Instead, we aimed to identify examples of evidence of effective social interventions which may promote mental health and well-being. This was intended to facilitate the identification of social policies which indirectly affected mental health.

We therefore began to map the evidence of effective interventions by using three main sources: the CRD *Wider Public Health Report *(*see*: ), which reviewed evidence from systematic reviews on wider determinants of health within a wide range of sectors (including the health sector); the (then unpublished) WHO report on Mental Health Promotion, which includes chapters on evidence of interventions and discussion of the mental health impact assessment of policies;[21] and Wilkinson and Marmot's Social Determinants of Health [18]. The most comprehensive of these was the CRD report, as it includes systematic reviews across all major sectors, and explicitly includes a chapter on mental health which lists social and service-level interventions with a summary of the evidence on their effectiveness and cost-effectiveness. There are many other sources, which, given time, could have been examined more systematically, but, as stated above, the intention was not to construct a comprehensive review of the effectiveness of social interventions to improve mental health.

From these sources, we identified examples of effective interventions within each of the broad categories of determinant. These were social or policy interventions for which there was some evidence that they would affect mental health and well-being. Again, this list does not claim to be comprehensive – compiling the evidence base for mental health could take many months (or perhaps years). Instead, this document was used as a pointer to the types of interventions which, within a particular category, may be effective. So, when reading a policy document, any policy, initiative or intervention which resulted from a policy, or which looked similar to the examples, or which appeared to fall within these categories, was noted. This was done in the next phase, the policy document analysis.

### 4. Policy document analysis

Next, policy documents were coded in order to capture two types of information: the degree of general alignment of policy 'interventions' with the framework (that is, what effect might this policy have on these determinants of mental health), and the degree to which there is any evidence for the potential impact of the policy on mental health and well-being. A short qualitative summary was also produced for each policy. This indicated, where possible, the extent to which the aims of the policy were aligned with the National Programme aims ("to raise awareness and promote mental health and well-being; to eliminate stigma and discrimination; to prevent suicide; and to promote and support recovery") and where specific interventions or commitments with the potential to impact on mental health were cited in the document.

### 5. In-depth interviews with policymakers

Interviews were sought with senior policymakers in the Scottish Government in order to learn more about policy formulation and the role of research evidence in this process. These were carried out in parallel with the documentary analysis. Semi-structured interviews were conducted with 19 senior policy makers using a semi-structured topic guide. The interviewees were identified through consultation with the study commissioners as key informants with respect to policy making in their area. Interviewees were located in the departments of Development, Education, Enterprise, Transport and Lifelong Learning, Health, Justice, and the Office of the Permanent Secretary. No Department refused to provide an interviewee, and most (17 of 19) of the interviewees were not known to the researcher. The interview topics included: cross-cutting policy making and mainstreaming of mental health and well-being, and barriers to these; the role of research evidence; the interviewee's awareness of the National Programme on Mental Health and Well-being, and its effects on different policy areas; the importance of mental health and well-being in different policy areas; and concepts of mental health and well-being. Not all of these issues are reported in this paper for reasons of space. Where permission was granted, interviews were tape-recorded and fully transcribed, or, where permission was withheld, permission to take detailed notes was sought. The transcripts and notes were then analysed thematically by one researcher (SP or SW), by categorising the interview data under the main topic headings. This approach used themes which were identified by the researchers *a priori *as being relevant to incorporating mental health into policy development, and others which emerged during the course of the interviews; the thematic structure was thus refined in the course of the analysis. The topic guide was not provided to interviewees in advance, and interviewees did not review the transcripts prior to analysis. Where permission was given, interviews were tape-recorded and fully transcribed; otherwise notes were taken with permission.

## Results

### (i) Policy document analysis

Most policy documents did not refer directly or indirectly to mental health, but most did refer at least indirectly to the *determinants *of mental health – for example, by describing how the policy may affect employment opportunities. In most cases, however, it was difficult to discern any research evidence base underpinning such statements and therefore difficult to assess the extent to which a particular policy was aligned with the goals of the National Programme. In a few documents there was explicit reference to research studies or government initiatives, which on theoretical grounds – or on the basis of previous research-have the potential to improve mental health.

In some cases, the lack of reference to evidence, or the lack of alignment between evidence and policy, simply reflects the different policy purposes of the documents; in others, however, there is more explicit use of and consideration of the need for evidence (see example 2, Table 3). Health Department documents, in particular, and an Education Department document "Being Well, Doing Well", which gave priority to the emotional well-being of children, made more explicit use of evidence.

**Table 3:** Examples of two policy documents with limited, and more extensive use of research evidence to support interventions

*1. Voluntary issues: The Scottish compact*

This document was an update of an earlier (1989) Scottish Compact document, which discussed issues such as protection of vulnerable youngsters, ending poverty and discrimination, and creating strong safe communities. While these aims, if achieved, would no doubt improve mental health and well-being, the document is largely written at an aspirational level, with little detail on specific interventions and how they may be implemented. In general terms, the descriptions of the importance of particular values – pluralism, diversity, and the rights of minorities – were consistent with the National Programme, but there was not enough detail to code or analyse this information. It was not possible therefore to identify or code examples of 'alignment' with the aims of the National Programme, simply because the document was written as a high-level policy document.
*2. Culture, arts and sport: Let's make Scotland more active*

This document was quite unusual in two respects: firstly, it used and cited examples of research evidence to make the case about the prevalence of mental ill-health; and, second, it also commented on the strength of that evidence. For example, the links between physical activity and academic achievement were described, but then acknowledged to be "not solid". The document also referred to the need to collect information on the costs and benefits of interventions. While specific details of interventions are often lacking (as is generally the case in policy literature), these examples suggest that the document is broadly concerned with the evidence base underpinning physical activity promotion and, in some cases, the mental health benefits which may result from this. There are also a few examples which address the mental health/well-being agenda. For example, while most of the risks of inactivity described in the document are in the physical domain, (such as obesity), physical inactivity is also linked explicitly to poor self-esteem, anxiety, and stress, as well as substance abuse and addictions. Although the direction of causality is unclear, the explicit linking of mental health with physical activity, and elsewhere the commitment to give equal value to social and emotional outcomes as well as physical health benefits, does suggest 'alignment', and an explicit acknowledgement of the underlying evidence.

In other cases policies may have had the potential to affect mental health, but the links were not made explicitly, or the document was written at such a general level that specific interventions were difficult to determine ("The Scottish Compact", for example, in Table 3). In the majority of cases (n = 12 of 20 documents) there may have been indirect effects on mental health, but these were not specified. The other eight documents showed some alignment with the goals of the National Programme and/or references to or use of use of research evidence.

In summary, for many of these policy documents the absence of specific descriptions of interventions made it difficult to determine whether a particular policy would, or would not, help promote the goals of the National Programme.

### (ii): Findings from interviews with senior policy makers

The themes from the interviews with the policy-makers fell into three broad categories: *cross-cutting policy and mainstreaming*, referring to collaborative efforts between government departments to develop strong, integrated policies for the broader public interest; *the role of research evidence*, referring to the need to collect, synthesise and apply research evidence across all stages of the policy cycle; and interviewees' recognition of the National Programme for Improving Mental Health and Well-Being. The first two of these are discussed below.

#### 1. Cross-cutting and mainstreaming

As noted earlier, cross-cutting policy is used in the Scottish Executive (and elsewhere) to refer to policies that deal with complex social issues by linking together difference departments, services and agencies. For example:

"Cross-cutting policy addresses issues irrespective of institutional or organisational boundaries. In this report it usually means policy developed jointly by Departments and which depends on a range of agencies for its delivery. Examples include social inclusion, improving health, rural development, tackling drugs, the New Deal and sustainable development."[14] This is also sometimes referred to as "joined-up" policy. Among interviewees there was a widespread commitment to the cross-cutting principle – that departments should work together to promote health and well-being. Many believed that its practice had increased, but enthusiasm was not found everywhere:

*" There are still pockets of resistance... I don't think it's that they aren't on board, that somehow they're ideologically opposed, it's just that it's sort of alien to them.... They're very focused on what they do and they don't really need or want to look outside" (Education/Pupil Support)*.

Political leadership and involvement of ministers were seen as key facilitators. However, the political system was felt to work against cross-cutting, with few, if any, rewards at the individual or departmental level for effective contributions to policy outcomes which 'belong' elsewhere in the Government.

The challenges and difficulties of implementing cross-cutting policy making were also recognised. Several conditions needed to be satisfied in order to maximise the effectiveness of the approach. First and foremost, political leadership was required:

"That absolutely underpins everything, you know, commitment of political leadership, engagement with the people out there who are going to have to work with the policy, absolutely critical at all stages, so they've got, a dreadful word, but that sense of ownership..." (Education/Lifelong Learning)

While some ministers wholeheartedly embraced that agenda, others were perceived to be more resistant:

*"... the principal barrier to cross-cutting partnership working is that ministers and other stakeholders ... aren't necessarily signed up to the idea of a unified whole and still bombard you with individual things that are only to do with your specific narrow remit." (Education/Pupil Support)*.

There were also many perceived disadvantages of the cross-cutting approach. For example, there appeared to be few, if any, rewards at the individual or departmental level for effective contributions to policy outcomes which 'belonged' elsewhere in the Government:

*"We haven't yet developed the accountability mechanisms that enable us to quantify and to account for the inputs that we make towards achieving non-cultural policy outcomes" (Education/Culture)*.

Moreover, the political system is structured in such a way that departmental identity and allegiance takes precedence over an inter-sectoral perspective:

*"... your bit of the organisation to deliver against certain objectives, getting your parliamentary questions answered, your ministerial correspondence done, providing briefing for your minister. And, you know, often these silo-orientated activities have to take precedence over the cross-cutting work" (Education/Sport)*.

While some departments might gain advantages from cross-cutting, through sharing responsibility for delivering on certain policy outcomes, others felt that they were having to find the resources to deal with an agenda that is not theirs:

*"Yes, the fact that everybody sees us as the dustbin. Do you know what I mean? The cross-cutting is almost ... an excuse, if you like, to dump work that's too difficult, or you don't have the funding, on somebody else, do you know what I mean? Dump a problem elsewhere" (Education/Lifelong Learning)*.

There was little evidence of support for policy proofing, defined as "the mechanism by which policies are assessed at design and review stages for their likely impact on particular areas of concern (e.g. poverty, equality, health)", as a separate stage in the policy cycle. Proofing was felt to be best incorporated into the policy development process via the provision of advice and guidance from colleagues from a mental health improvement perspective.

#### 2. The role of research evidence

The majority of interviewees acknowledged the importance of collecting, synthesising and applying research evidence across all stages of the policy cycle. While there was general appreciation of the value of quantitative data, and for syntheses of evidence (*"Because of the huge variety of issues we deal with, I prefer summaries or meta-analyses of evidence that can give an overview of a particular field.": Health Improvement*) considerable support was expressed for qualitative and person-focused research. The power of the latter to make the data 'come alive' or construct a convincing narrative was emphasised by several interviewees, for example:

*"...the literature on the potential health benefits of universal free school meal provision [commissioned by the Department] ... draws a distinction between things that were sort of rigorous and quantitative and then softer stuff. But, quite frankly, the stuff I found most valuable was the "softer" stuff. It was perceptions about how the system works and how people engage with the idea of health and what sort of investment people put into health benefits" (Education/Pupil Support)*.

Some interviewees drew attention to the lack of relevant and important information or evidence in their policy area. Others, however, felt that, while there was no shortage of data on the scale of the (policy) problem ('what is'), convincing approaches to solving the problem ('what is to be done') were often conspicuous by their absence:

*"You'll find there's masses and masses of research evidence which describes problems and there's not an awful lot of it that takes the next step and looks at what kind of remedies, what kind of solutions does all of this point to. We're not short of people telling us ... what problems are.... Sometimes we're a bit short of people telling us what we could actually do" (Development/Social Justice)*.

Waiting for evidence could also stifle innovation:

*"If you always have to have evidence, then you'll never do anything a bit different"(Health Improvement)*.

When evidence on "what works" was absent, other types of applied evidence needed to be used, though a distinction seemed to be drawn by one respondent between clean, academic, scientific evidence, and "dirtier" policy or practice-oriented research:

*"I know that's "dirty" research...very policy or practice-oriented research. But I suppose that's the kind of research that I'd find most helpful"*. (Social Justice)

Overall, the interviews found much support among policymakers for integrated policymaking – that is, working between departments to ensure that the positive impacts of policies were enhanced. However, it also identified a need for greater awareness of the societal and policy determinants of mental health and well-being. As a result of the analysis of the interviews we identified a number of recommendations for the National Programme to enhance the mainstreaming of mental health issues in different policy areas, which are summarised elsewhere .

Additional file 1 provides a short summary of the project findings.

## Discussion and conclusion

It is often argued that producing healthy public policy depends on effective intersectoral working between government departments, along with better use of research evidence to identify positive and negative impacts of policies. This study piloted an approach to rapidly appraising the mental health effects of mainly non-health sector policies, drawing on both theoretical understandings of mental health and its determinants, and, where possible, its evidence base. It drew on policy documents as a source of information, and in-depth qualitative interviews, as recommended elsewhere [23].

Our experience suggested, however, that this approach may be of limited use for 'public' policy documents, other than to provide a very broad description of orientation or alignment. Part of the problem lies with the use of policy documents for scientific purposes, for which they are clearly not intended; policy language often does not easily lend itself to the type of systematic appraisal we attempted, not least because explicit references to research evidence are of course the exception rather than the norm in policy documents. For example, some of these documents use suggestive, and sometimes metaphorical language to suggest new directions, or support for particular ideas, but without making specific commitments.

By contrast, as expected, the qualitative interviews were able to explore underlying issues in greater detail. These data illustrate the reality of day-to-day working which underlies the broader policy goals and statements – for example the lack of incentives to mainstreaming, limited understanding of mental health terminology, and structural problems such as lack of time and resources. This partly explains why the identification of an 'evidence base' underpinning policy statements cannot often be discerned. Simple lack of evidence on mental health inequalities may also be a reason [24,25].

Variable use of evidence also reflects variability in understanding of the wider determinants of physical and mental health. Broadly speaking, the greatest understanding is demonstrated in documents which emerge from the health sector, or which discuss health-related issues (such as health behaviours). One implication of this is that appraising policies for health outcomes (not just mental health outcomes) may have an inherent bias against non-health sector policies. Policy documents which originate from (or close to) the health sector are probably well-used to communicating the importance of health, well-being and social inclusion. Other sectors, however, are likely to be focused on priority outcomes other than health; alternatively, they may be focused on the same outcomes, but these may be expressed differently. These valid outcomes of policies may be easily overlooked, or not given sufficient importance, though they are undoubtedly important in influencing public health.

There are other possible biases. One is the existence of an 'optimistic bias' [26] in policy documents: that is, there is an in-built bias towards reporting potentially positive impacts and overlooking negative impacts (including costs).[27] There is also an 'availability bias', relating to the availability of evidence [26,28]. The degree of 'alignment' – or not – is based on statements in the document, but these statements may mainly reflect the amount and availability of the existing evidence, rather than the extent to which it is actually used for policy purposes. For example there is still relatively little evidence on the impacts of transport policies on mental health and well-being [29,30]. Alignment, or apparent lack of it, may thus reflect the maturity of the evidence-based approach in different policy sectors or research fields, rather than policy disinterest in evidence or in mental health and well-being. However it may also simply reflect the fact that policies outside the health sector do not consider incorporating impacts on health when formulating policy; for example, the primary purpose of a new transport policy is more likely to be economic improvement rather than health improvement.

The interviews with policy makers were more fruitful, and suggested that there is considerable support for integrated policy making. Nevertheless, there were still "pockets of resistance" and several potential sources of threat to implementing such an approach, including (lack of) political leadership and ministerial engagement, insufficient rewarding of cross-cutting work, the persisting influence of traditional departmental structures and boundaries, and scarcity of resources. This is echoed in one of the conclusions of a recent report to the WHO Commission on the Social Determinants of Health from the Measurement and Evidence Knowledge Network. This report noted that intersectoral collaboration may require the support of health champions to help other sectors understand why they should get involved in tackling health and health inequalities [31].

Despite recognising the restricted influence of research on the policy process, the role of 'evidence' at all stages of the policy cycle was widely acknowledged, with enthusiasm expressed for the use of qualitative, as well as quantitative, data collection methods. Some interviewees did express the need for more information in their policy area. For others, however, the lack of evidence concerning what is to be done (problem solving) was of much greater concern.

Finally, healthy public policy depends on effective intersectoral working between government departments, along with better use of research evidence to identify policy impacts. In the case of the social determinants of health, an evidence-based approach to policymaking and to policy appraisal may however require drawing as much upon existing theoretical frameworks as upon research evidence about the effects of interventions [32-36].

## Competing interests

The authors declare that they have no competing interests.

## Authors' contributions

SP, MP and AM conceived the study and were responsible for its design and coordination. SW and ST conducted the documentary analysis and majority of interviews; the remaining interviews were conducted by SP. MP and SP drafted the manuscript. All authors read and approved the final manuscript.

## Pre-publication history

The pre-publication history for this paper can be accessed here:



## Supplementary Material

Additional file 1 This is a short summary in pdf format of the project findings requested by the project funders. Summary report on Mental Health Improvement Project findings. Click here for file
