# Supplementary material for: "We're not short of people telling us what the problems are. We're short of people telling us what to do": An appraisal of public policy and mental health
Source: BMC Public Health. 2008 Sep 15;8:314. doi: 10.1186/1471-2458-8-314 (PMC2561038; doi:10.1186/1471-2458-8-314)
Supplement: Additional file 1 — This is a short summary in pdf format of the project findings requested by the project funders. Summary report on Mental Health Improvement Project findings. [file 1471-2458-8-314-S1.pdf]

MENTAL HEALTH IMPROVEMENT

An appraisal of Scottish policy

In 2004 NHS Health Scotland and the Scottish Executive's National Programme for Improving Mental Health and Well-Being commissioned a review of Scottish policy with respect to mental health improvement. This project involved mapping out the key policy areas in Scotland, gathering key policy documents from these areas to identify references to mental health improvement, and assessing the degree to which current policy and the research evidence are aligned. Interviews were also conducted with key policy makers from six Scottish Executive departments, to explore the impact of the National Programme and the importance of mental health and well-being in different policy areas.

KEY POINTS

- Most Scottish Executive policy documents refer indirectly to determinants of mental health and well-being. However, in most cases, information on the research evidence underpinning such statements is not provided and, with the exception of health sector policies, the potential effects of policies on population mental health are often unrecognised.
- There is much support for integrated policymaking offering solid foundations for progress. However, there are many challenges to cross-cutting and mainstreaming. A main difficulty is that the political system is structured in such a way that departmental activity, identity, performance and allegiance can take precedence over an inter-sectoral perspective.
- There is little support for policy proofing as a separate stage in the policy cycle. However, advice and guidance from a mental health improvement perspective, provided at the policy development stage, is likely to have greater impact and receive more support.
- Despite recognising the restricted influence of research on the policy process, the need for research evidence is acknowledged and accepted.
- Understanding of 'mental health' and related concepts varies both within and between Executive departments. For some the area is equated with mental disorders or problems and related legislative and service issues, while for others it is interpreted more broadly, recognising the role of positive aspects of well-being, such as personal and social skills, confidence and self esteem.
- Awareness of the National Programme also varies within and between different departments. In some sectors there is no knowledge of the Programme, while in others there is definite evidence of a solid foundation on which to build more integrated, cross-cutting policy.
- To enhance the mainstreaming of public mental health the National Programme should: increase awareness in non-health policy areas of the public health approach to mental health; offer customised support and direct input to other departments and develop closer interpersonal links; and consider ways of demonstrating an understanding of other departments' agendas and priorities.

1 BACKGROUND

Mental health and well-being is essential for the country's social and economic well-being. However, improving mental health at a population level requires a cross-cutting policy agenda: healthy public policy is the responsibility of many, if not all, departments. No single public sector or government department is alone responsible for good mental health (see **Box 1**).

Improving mental health involves:

- promoting mental health in the whole population (e.g. self esteem and confidence, feelings of belonging, coping skills, resilience, etc)
- preventing mental health problems, and
- improving the quality of life of people experiencing mental health problems.

These are three integral components of the Scottish Executive's agenda for health improvement. In October 2001 the National Programme for Improving Mental Health and Well-Being was launched to raise the profile of, and to support further action in, mental heath improvement. In 2004 NHS Health Scotland and the National Programme commissioned an appraisal of Scottish Executive policy, to assess the degree of 'alignment' between Scottish Executive policy and the goals of the National Programme. The results of that study are reported below.

2 THE STUDY

The study involved two components: an analysis of Scottish Executive policy documents and in-depth interviews with key policy makers.

Documentary analysis

The main objectives of this part of the project were to:

- map out key policy areas in Scotland
- gather key policy documents and identify references to mental health improvement
- identify evidence-based links with mental health improvement
- assess the degree to which current policy and evidence are aligned; and
- summarise key points from the policy documents.

The list of policy documents selected for analysis was agreed with commissioners in July 2004. The documents covered education, social inclusion, enterprise, transport and life long learning, health, justice, equalities, community regeneration, homelessness, housing, voluntary issues, rural development, and culture, arts and sport (see **Box 2**). Documents were selected according to the degree of connectedness to the key aims of Scottish Executive policy making in general (the encouragement of a smart, successful Scotland, closing the opportunity gap and giving every child and family the best start) and the aims of the National Programme more specifically.

BOX 1

Societal determinants of mental health

- Addictions/substance dependence.
- Cultural values.
- Culture and leisure facilities.
- Economic resources and welfare.
- Education policies.
- Health and social services.
- Housing and neighbourhood.
- Justice.
- Physical health.
- Societal structures and resources.
- Transport.
- Workplace policies.

BOX 2

Scottish Executive policy documents: the sample (government department/area shown in brackets)

- A Scotland where everyone matters (social inclusion).
- Being well – doing well (education).
- It's everyone's job to make sure I'm all right (education).
- For Scotland's children (education).
- A smart, successful Scotland (enterprise, transport, lifelong learning).
- The way forward: framework for economic development in Scotland (enterprise, transport, lifelong learning).
- Life through learning through life (enterprise, transport, lifelong learning).
- Scotland's transport (enterprise, transport, lifelong learning).
- National plan on alcohol problems (justice).
- Towards a healthier Scotland (health).
- Improving health in Scotland – the challenge (health).
- The equality strategy (equalities).
- Race equality scheme (equalities).
- Community regeneration statement (community regeneration).
- Homelessness task force final report (homelessness).
- Housing improvement task force (housing).
- Scottish compact (voluntary issues).
- Cultural policy statement (culture, arts and sport).
- Let's make Scotland more active (culture, arts and sport).
- Rural Scotland: A new approach (rural development).

To guide the analysis, a framework outlining the key determinants of population mental health was devised based on the categories of mental health determinants listed in **Box 1**. Each document was read in detail and relevant information extracted to a template which was based on these categories. These templates were then coded and summarised in order to capture two types of information: the degree of general alignment of policy 'interventions' with the framework (that is, what effect might this policy have on these determinants of mental health?), and the degree to which there is evidence for potential impact of the proposed policy on mental health and well-being. A short qualitative summary was then produced for each policy.

Interviews with policy makers

Fourteen in-depth, semi-structured interviews were conducted with 19 senior policy makers. The National Programme identified interviewees as key informants with respect to policy making in their area. **Box 3** lists the range of departments within which interviewees were located. **Box 4** lists the main topics covered in these interviews.

3 KEY FINDINGS

(i) Appraisal of Scottish Executive policy documents

Most policy documents did not refer directly or even indirectly to mental health, but most did refer indirectly to the *determinants* of mental health – for example, by describing how the policy may affect employment opportunities (which will in turn affect mental health). In most cases it was difficult to discern any evidence base underpinning such statements, and in most cases it proved difficult to assess the extent to which a particular policy was aligned with the goals of the National Programme, mainly because evidence was not cited.

Some policy documents, however, did appear to be well-aligned – Health Department documents, for example, and *Being Well, Doing Well* (Education Department), which gave priority to the emotional well-being of children. Again, other documents which spoke of reducing stigma and promoting social inclusion (for example, *A Smart, Successful, Scotland* and *Learning through Life*) would be expected to contribute to the promotion of better mental health. Other policy areas have the potential to do so, but either the links were not made explicit, or the document was written at such a 'high' or general level that specific actions or interventions were difficult to determine (the *Scottish Compact* and the *Cultural Policy Statement*, for example). In others, there may well be indirect positive effects on mental health (for example, from Rural Development, as outlined in *Rural Scotland: A new approach*), but these were not specified.

In summary, for many Scottish Executive policy documents, the absence of specific descriptions of interventions makes it difficult to determine whether a particular policy would or would not help promote the goals of the National Programme. Opportunities to link the policy more clearly to the determinants of health and well-being are very often missed, and there is generally no reference to relevant evidence (if it exists).

It should be noted, however, that the appraisal of policy documents does have several limitations and possible biases:

- The analysis of non-health policy documents may underestimate the real, but indirect, positive or negative effects of those policies on mental health.
- Policy documents are not academic articles, which makes the identification and analysis of evidence in those documents difficult.
- 'Absence of evidence' of policy impacts does not equate to 'evidence of absence'.
- Evidence of 'alignment' with the evidence base does not constitute robust evidence that the policy itself will have any impact on health; this requires evaluation of the actual impact of policies.

BOX 3

Interviews were conducted with 19 senior policy makers from the following departments

- Development.
- Education.
- Enterprise, transport and lifelong learning.
- Health.
- Justice.
- Office of the Permanent Secretary.

BOX 4

Interview topics

- Cross-cutting policy making and mainstreaming, and barriers to these.
- The role of research evidence.
- Awareness of the National Programme, and its effects on different policy areas.
- The importance of mental health and well-being in different policy areas.
- Barriers to mainstreaming mental health in different policy areas.
- Concepts of mental health and well-being.

- The use of different language can result in the outcomes of polices being overlooked; for example, appraising policy documents for health outcomes may introduce a bias against non-health documents.

(ii) Findings from interviews with senior policy makers

Cross-cutting and mainstreaming

In this context 'cross-cutting policy making' refers to collaborative effort between government departments to develop strong, integrated policies for the broader public interest. Among interviewees there was a widespread commitment to the cross-cutting principle and many believed that its practice had increased since devolution. However, enthusiasm was not found everywhere:

> *'There are still pockets of resistance… there are bits, [that are] very focused on what they do and they don't really need or want to look outside.'*

Political leadership and involvement of ministers were seen as key facilitators. However, the political system was felt to work against cross-cutting, with few, if any, rewards at the individual or departmental level for effective contributions to policy outcomes which 'belong' elsewhere in the Executive:

> *'We haven't yet developed the accountability mechanisms that enable us to quantify and to account for the inputs that we make towards achieving non-[departmental] policy outcomes.'*

> *'Your bit of the organisation [has] to deliver against certain objectives, getting your parliamentary questions answered, your ministerial correspondence done, providing briefing for your minister. And, you know, often these silo-orientated activities have to take precedence over the cross-cutting work.'*

Mainstreaming, too, was challenging: *'There's no short answer, it has got to be planned rigorously and meticulously and resources have got to follow, otherwise it won't happen'*. Policy proofing (for example, mental health proofing) could help with mainstreaming, but there was the risk that it could degenerate into a tick the box exercise. There was therefore little support for policy proofing as a separate stage in the policy cycle. Proofing was felt to be best incorporated into the policy development process via the provision of advice and guidance from colleagues from a mental health improvement perspective.

The role of research evidence

The majority of interviewees acknowledged the importance of collecting, synthesising and applying research evidence across all stages of the policy cycle. Some felt that, while there was no shortage of data on the scale of the problem, convincing solutions were conspicuous by their absence:

> *'You'll find there's masses and masses of research evidence which describes problems and there's not an awful lot of it that takes the next step and looks at what kind of remedies, what kind of solutions does all of this point to. We're not short of people telling us … what problems are. Sometimes we're a bit short of people telling us what we could actually do.'*

Concepts of health and well-being

Interviewees were asked about their understanding of the concepts 'mental health', 'mental health improvement', 'mental health promotion', 'mental well-being' and 'emotional health'. There was considerable variation in how these were interpreted. For some, this meant community well-being and community capacity, and in some cases (the role of sport, for example) there was a strong emphasis on the positive aspects of mental health. This was in contrast to other interviewees who assumed that the focus of the question was on mental *disorder* or *'the medical end of mental health…'* in the words of one respondent. Many policy areas seem to assume a service model of mental ill-health (with treatment targeted at individuals), with little awareness of the public (mental) health model of mental health, with its emphasis upon prevention and promotion at community, workplace and societal (as well as individual) levels.

Recognition of the National Programme

Interviewees' knowledge of the National Programme was equally variable: at one extreme, there was evidence of familiarity and awareness and close working relationships; at the other, limited awareness, and absence of connections to the programme.

Overall, this study found much support among policymakers for integrated policymaking. However, it also identified a need for greater awareness of the societal determinants of mental health and well-being, and a need for greater consideration of the mental health impacts of Scottish Executive policies.

Implications for the National Programme

Recommendations for the National Programme to enhance the mainstreaming of mental health issues in different policy areas included:

- increasing awareness in non-health policy areas of the public health approach to mental health, with its emphasis on prevention and promotion at individual, community and societal levels
- identifying ways of demonstrating an understanding of other departments' agendas and priorities
- developing more customised, individualised approaches to other departments, concentrating most on those which are charged with delivery
- developing closer interpersonal links at appropriate levels; and
- where written guidance is provided, ensuring that products are short and attention is paid to readability.

Contacts for further information

The research described in this short report was commissioned by NHS Health Scotland in 2004 and was conducted by: Stephen Platt[1], Mark Petticrew[2], Allyson McCollam[3], Sarah Wilson[1] and Sian Thomas[2].

To obtain a copy of the full report, please contact Emma Hogg, at emma.hogg@health.scot.nhs.uk

1. Research Unit in Health, Behaviour and Change (RUHBC), University of Edinburgh, EH8 9AG
2. MRC Social and Public Health Sciences Unit, University of Glasgow, G12 8RZ
3. Scottish Development Centre for Mental Health, 17a Graham Street, Edinburgh EH6 5QN

Published by Health Scotland.

Edinburgh Office: Woodburn House, Canaan Lane, Edinburgh EH10 4SG

Glasgow Office: Clifton House, Clifton Place, Glasgow G3 7LS

© NHS Health Scotland, 2005.

Health Scotland is a WHO Collaborating Centre for Health Promotion and Public Health Development.

healthyliving

NHS Health Scotland

2209 7/2005
